# Prefrontal Activation is Associated With Gait Quality During an Attentional Task in Older Adults

**DOI:** 10.1093/geroni/igab046.3521

**Published:** 2021-12-17

**Authors:** Emma Baillargeon, Anisha Suri, Nemin Chen, Xiaonan Zhu, Caterina Rosano, Ervin Sejdic, Theodore Huppert, Andrea Rosso

**Affiliations:** 1 University of Pittsburgh, Pittsburgh, Pennsylvania, United States; 2 Swanson School of Engineering, University of Pittsburgh, Pittsburgh, Pennsylvania, United States

## Abstract

Prefrontal cortical activation varies by walking task and is a marker of attentional demand. We compared prefrontal activation by functional near-infrared spectroscopy (fNIRS) to accelerometry-derived gait quality. We hypothesized greater activation with lower gait quality (greater step-time coefficient-of-variation, decreased cadence, smoothness, regularity, and signal variability). Participants (n=114; age 74.4±6.0 years, 59.6% female) were independently ambulating individuals >64 years. Attentional (reciting every-other alphabet letter) and physical (uneven surface) challenges mimicked community mobility and provided four 15m walking conditions: even, uneven, ABC-even, and ABC-uneven. fNIRS data were referenced to quiet standing and averaged within left and right hemispheres. Gait metrics from a tri-axial accelerometer at the lower-back included cadence (steps/min), step-time coefficient-of-variation, signal variability (standard deviation), smoothness (harmonic ratio), and regularity (entropy). Associations between fNIRS and gait were quantified using Pearson correlations (α=0.05). Results were consistent across hemispheres, gait axes, and robust to adjustment for age and gait speed; we report unadjusted coefficients for left hemisphere and anterior-posterior gait direction. Greater prefrontal activation was associated with slower cadence (r=-0.220, p=0.019), lower signal variability (r=-0.228, p=0.015), and reduced smoothness (r=-0.194, p=0.039) during ABC-even. No relation was observed for step-time coefficient-of-variation or regularity. Results were similar for the ABC-uneven condition, except there was no association with gait smoothness but was with step-time coefficient-of-variation (r=0.25, p=0.007). Prefrontal activation was not correlated to gait quality in non-ABC conditions. Our findings support our hypothesis only during the ABC challenge, suggesting that older adults may rely on prefrontal activation to complete attentional but not physical challenges during gait.

